# Diffusion-based tractography atlas of the human acoustic radiation

**DOI:** 10.1038/s41598-019-40666-8

**Published:** 2019-03-11

**Authors:** Chiara Maffei, Silvio Sarubbo, Jorge Jovicich

**Affiliations:** 1 Athinoula A. Martinos Center for Biomedical Imaging, Massachusetts General Hospital and Harvard Medical School, Charlestown, USA; 2 Center for Mind/Brain Sciences - CIMeC, University of Trento, Rovereto (TN), Italy; 3 Division of Neurosurgery, Structural and Functional Connectivity Lab (SFC-LSB) Project, “S.Chiara” Hospital, Trento APSS, Italy; 4 Department of Psychology and Cognitive Sciences, University of Trento, Trento, Italy

## Abstract

Diffusion MRI tractography allows *in-vivo* characterization of white matter architecture, including the localization and description of brain fibre bundles. However, some primary bundles are still only partially reconstructed, or not reconstructed at all. The acoustic radiation (AR) represents a primary sensory pathway that has been largely omitted in many tractography studies because its location and anatomical features make it challenging to reconstruct. In this study, we investigated the effects of acquisition and tractography parameters on the AR reconstruction using publicly available Human Connectome Project data. The aims of this study are: (i) using a subgroup of subjects and a reference AR for each subject, define an optimum set of parameters for AR reconstruction, and (ii) use the optimum parameters set on the full group to build a tractography-based atlas of the AR. Starting from the same data, the use of different acquisition and tractography parameters lead to very different AR reconstructions. Optimal results in terms of topographical accuracy and correspondence to the reference were obtained for probabilistic tractography, high b-values and default tractography parameters: these parameters were used to build an AR probabilistic tractography atlas. A significant left-hemispheric lateralization was found in the AR reconstruction of the 34 subjects.

## Introduction

Diffusion-based MRI tractography allows *in-vivo* and non-invasive characterization of white matter architecture of the human brain. Since the introduction of the diffusion MRI tensor model^1^, one of its major applications has been the localization and description of white matter fibre bundles in the brain^2,3^. Most of these well-known bundles correspond to the associative fibre pathways that, coursing the longitudinal plane, connect different cortical regions of the brain. However, these major bundles represent the brain’s “highways” and constitute only a partial sample of all the white matter connections, the full architecture of which is sensitively more complex and unknown.

The introduction of more advanced multi-fibre-based diffusion models has helped improving the topographical knowledge of these and other main white matter pathways^4^. However, some primary bundles are still only partially reconstructed, or not reconstructed at all^5^. The acoustic radiation (AR) represents an example of such an omission in tractography studies. This bundle constitutes a primary sensory pathway conveying auditory information from the medial geniculate nucleus (MGN) of the thalamus to the auditory cortex on the transverse temporal gyrus of Heschl (HG)^6,7^. Because of its anatomical features, the AR goes undetected when using the diffusion tensor model^4,8^. In a previous human brain *post-mortem* dissection study from our group^9^ we showed that the AR fibres are characterized by a fully transversal course from the mid-line to the cortex, along which they cross some of the major fibre systems of the brain. This topographical feature, with its severe fibre crossing, has largely prevented the investigation of its anatomy in humans *in-vivo*. At present, most studies investigating the correlation between auditory pathways and auditory deficits employ region of interest (ROI) analyses^10^; however ROI-based analyses lack anatomical specificity and do not cover the whole extent of the pathway of interest. Tractography studies investigating language comprehension networks haven’t included the AR; however this primary sensory bundle is responsible of inputting the auditory information into the system and it is probably involved in the first steps of language processing^11^. The successful and reliable *in-vivo* reconstruction of this bundle using diffusion-based tractography techniques may allow for the exploration of the morphology and topography of these fibres in humans, and for the correlation with anatomical and functional aspects of audition and language.

The combination of ultra-high b-values and probabilistic tractography showed that results comparable to *post-mortem* Klinger’s dissections can be obtained *in-vivo*^9^. However, acquiring such high quality diffusion MRI data can be challenging, in particular with the constrains of typical clinical settings, which are commonly consistent with single shell, lower b-values MRI diffusion acquisition protocols. It is therefore important to understand how single b-value MRI acquisition and tractography analysis choices affect the reconstruction of the AR.

The final result of any MRI-derived tractography reconstruction intimately depends on both the acquisition and the tractography parameters. Different studies have investigated how changing analysis parameters, such as angular resolution^12,13^, spatial resolution^12^, tractography algorithm^14^, and tractography parameters^15–19^ affects the reconstruction of well known white matter tracts. However, as no one-for-all solution exists in diffusion tractography, the optimal parameters depend on the specific bundle under investigation. It remains thus unclear how these parameters affect the tractography reconstruction of the AR.

In this study, we systematically investigated the effects of MRI acquisition and tractography parameters on the diffusion-based tractography reconstruction of the AR using data publicly available from the MGH-USC Human Connectome Project. The main aims of this study are: i) using a subgroup of subjects and a reference AR for each subject, define an optimum set of MRI acquisition and tractography parameters for the reconstruction of the AR by systematically characterizing AR reconstruction effects as function of several parameters (MRI acquisition: b-value and number of diffusion encoding directions; tractography algorithm: probabilistic and deterministic; tractography parameters: angle threshold and step size), and ii) use the optimum parameter set on the full group of subjects to characterize the AR and build a tractography-based atlas of the AR.

## Results

### Acoustic Radiation: Optimal tractography reconstruction

**Figure 1:**
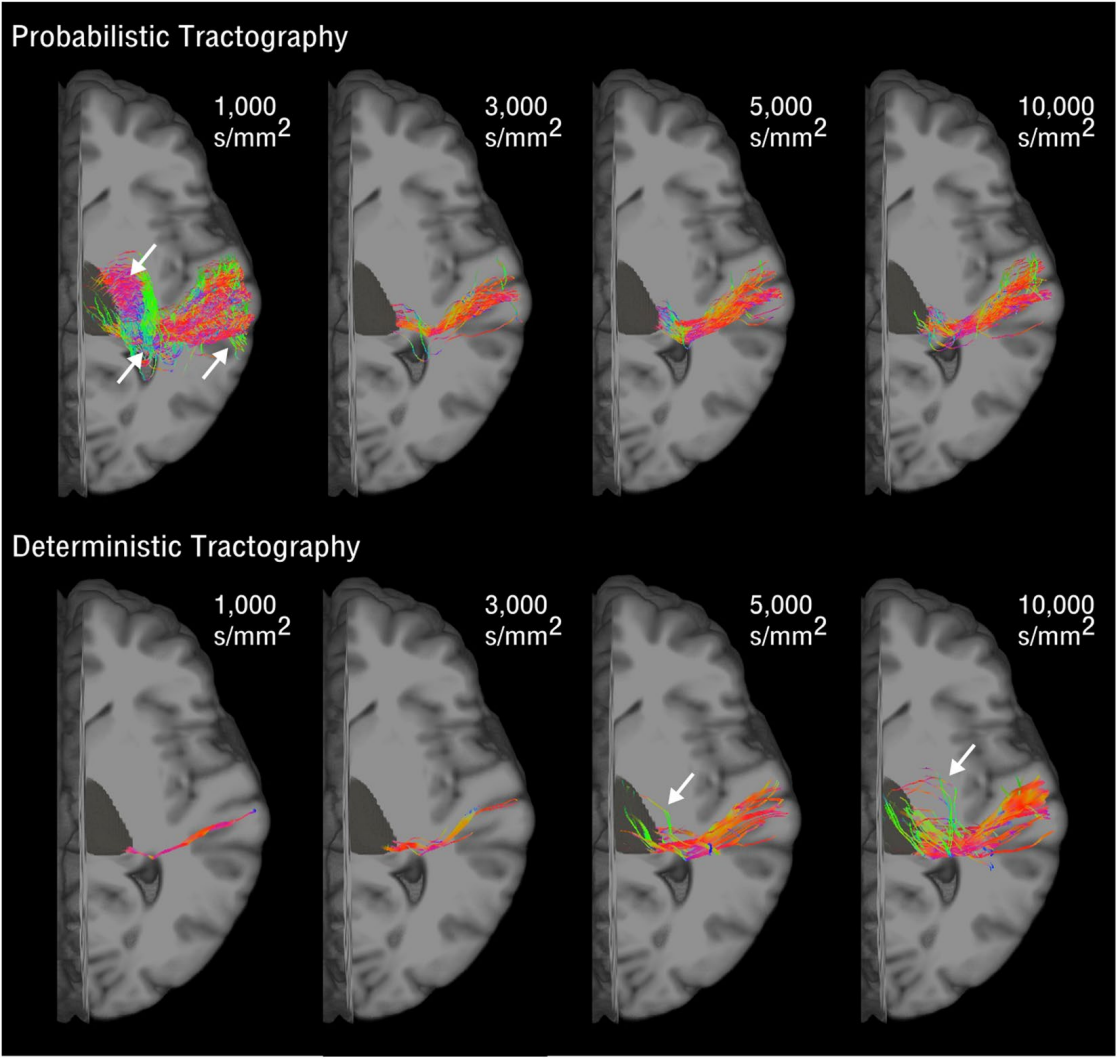
Diffusion MRI Acquisition Parameter Effects on Acoustic Radiation Reconstructions. The image shows the probabilistic (upper row) and deterministic (lower row) tractography reconstruction of the acoustic radiation in the right hemisphere. A representative subject is shown for each diffusion b-value shell and default tractography parameters (angle = 45°, step size = 0.75 *mm*). White arrows point to false positives in the tractography reconstructions. The reconstructed streamlines are shown overlaid on the T1-weighted image of the subject. The streamline colour encodes directionality (anterior posterior: green, superior-inferior: blue, left-right: red). The 3D rendering of the right thalamus is also shown in dark grey.

**Figure 2:**
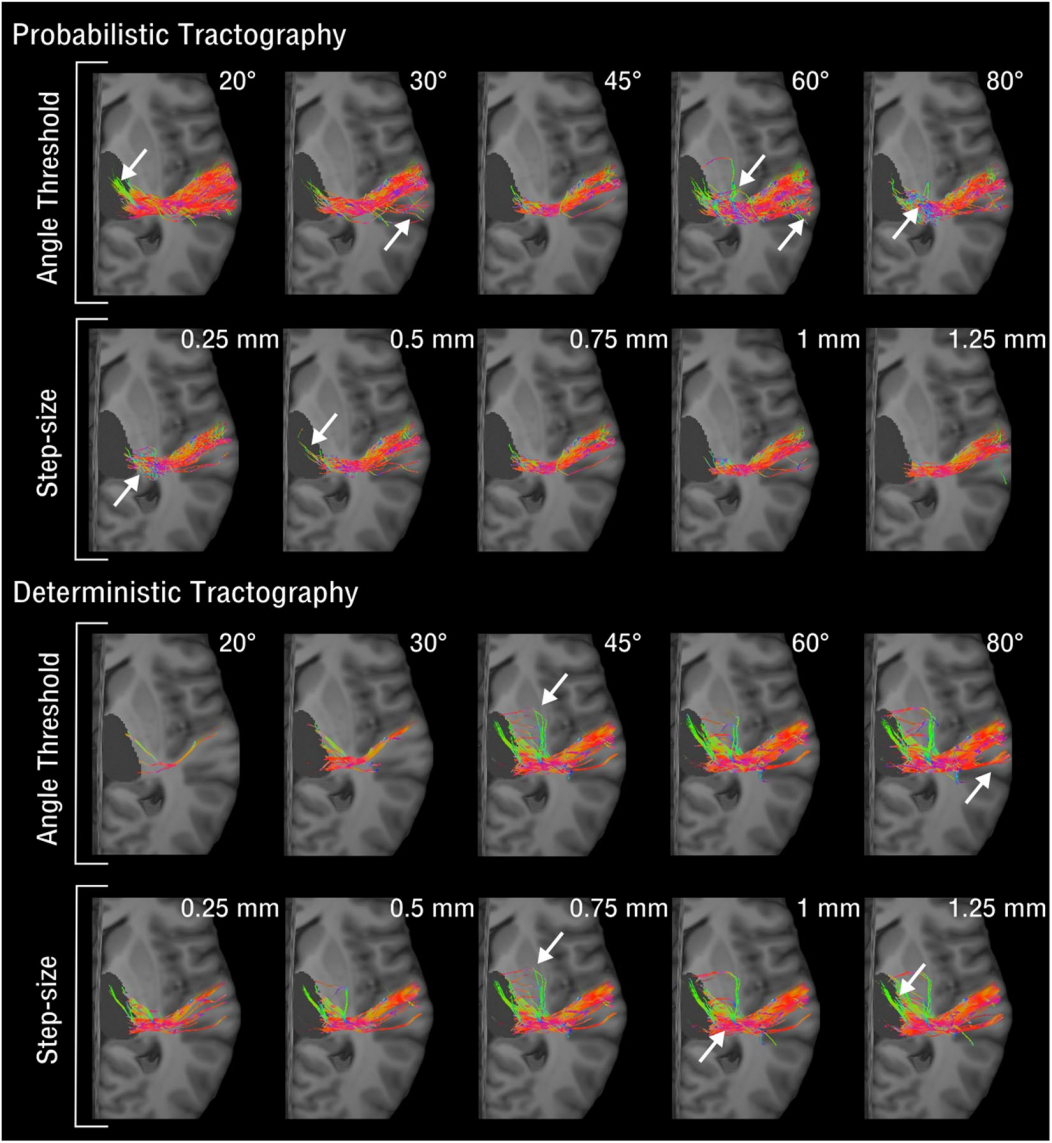
Tractography Parameters Effects on the Acoustic Radiation Reconstruction. The image shows the effects of tractography parameters (angle threshold and step size) on the reconstruction of the right hemisphere acoustic radiation for both probabilistic and deterministic algorithms on a representative subject using an MRI diffusion acquisition with b = 10,000 *s*/*mm*^2^. White arrows point to false positives in the tractography reconstructions. The reconstructed streamlines are shown overlaid on the T1-weighted image of the subject. The streamline colour encodes directionality (anterior posterior: green, superior-inferior: blue, left-right: red). The 3D rendering of the right thalamus is also shown in dark grey.

In a subgroup of 5 subjects we first evaluated how tractography reconstructions of the acoustic radiation (AR) were affected by the choice of the MRI shell (*b* = 1,000; 3,000; 5,000; 10,000 *s*/*mm*^2^), the tractography algorithm (probabilistic *versus* deterministic), and the tractography parameters (angle threshold and step size) (Section Optimal AR tractography reconstruction). We found that the choice of parameters notably affected the AR reconstructions. Figures 1 and 2 illustrate these effects on the right hemisphere AR in one of the 5 subjects.

For the lowest b-value (b = 1,000 *s*/*mm*^2^) and default parameters, the deterministic reconstruction provided almost no AR streamlines in all 5 subjects. On the contrary, the probabilistic methods resulted in a great amount of streamlines reconstructed between the thalamus and the Heschl’s gyrus (HG) (Fig. 1). Many of these streamlines, however, likely constitute false positive reconstructions, resulting from the intersection of the AR with other major bundles coursing the vertical axis of the medial brain. For higher b-values (>*b* = 3,000 *s*/*mm*^2^) deterministic reconstructions increasingly provided more AR streamlines, and probabilistic reconstructions showed less false positive streamlines on the vertical plane. At the highest b-value (*b* = 10,000 *s*/*mm*^2^), the AR reconstructions of probabilistic and deterministic methods tend to converge to similar anatomical representations (Fig. 1).

When observing the effects of tractography parameters (Fig. 2), the probabilistic approach resulted overall more robust than the deterministic one. The angle threshold differently affects the AR reconstructions at all b-values and for both approaches, with a stronger effect on deterministic reconstructions: for low angle thresholds (20°) no, or very few, streamlines are reconstructed even at high b-values. For probabilistic tractography and b = 1,000 *s*/*mm*^2^ the volume of the tract strongly decreases for angle = 20°, probably reflecting the elimination of most artefactual loops. Overall, the step size does not have a strong impact on the reconstructions at any of the different shells, for tic and probabilistic tractography.

### Acoustic radiation: Anatomical evaluation

In order to determine an optimal acquisition and analysis parameter set for the AR reconstruction we computed the Dice coefficient of spatial overlap between each tractography estimate and a manually filtered reference AR. This reference AR was built for each subject as described in the Methods section (AR dissection protocol).

**Figure 3:**
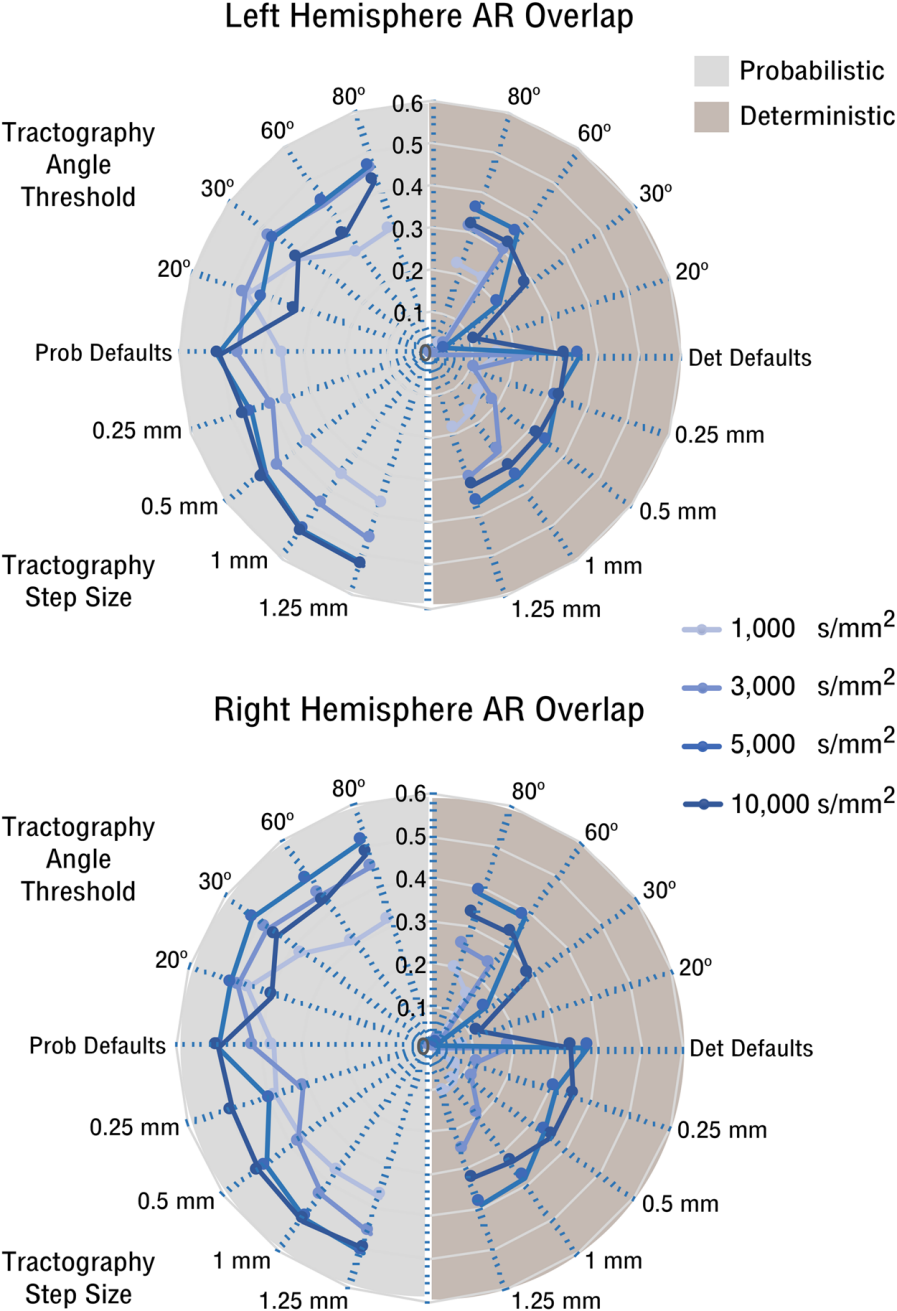
Acoustic Radiation Topographical Evaluation. Left/right hemisphere radar plots of the mean Dice coefficient of spatial overlap between the acoustic radiation tractograms estimated using different acquisition and analysis parameters relative to a reference acoustic radiation manually edited for each subject. Plots show mean Dice results from four subjects. The Dice coefficient is plotted for the two algorithms (probabilistic and deterministic) and for the different angle and step size parameters at the four shells (indicated by different line colours).

Figure 3 shows the group summary results of the Dice coefficient of spatial overlap (AR reconstruction relative to reference AR) for both acquisition and tractography parameter manipulations. The pattern of spatial overlap looks very similar across the two hemispheres. Dice coefficient resulted overall higher for probabilistic reconstructions than for deterministic ones, and higher when using higher b-values. For probabilistic tractography, increasing the angle decreases the overlap for high b-values, but increases the overlap for low b-values. At b = 1,000 *s*/*mm*^2^, for angle = 20° the overlap coefficient is very close to the reconstruction of the AR at high b-values (Fig. 3). For all shells the overlap slightly increases when bigger step sizes are used. For the deterministic reconstructions the overlap is strongly affected by the angle, increasing for higher angle values for all shells, while greater step sizes only slightly affects the reconstruction with no clear trend.

For each algorithm (probabilistic and deterministic) we used a non-parametric Kruskall-Wallis test to evaluate whether varying b-value, step size, and angle threshold affected the spatial overlap of the tractograms with the reference AR. Significant effects (*α* = 0.05) were found only for deterministic tractography, for acquisition parameters (*p* = 0.0127) and angle threshold (*p* = 0.0327).

Overall, the high b-value shell, the probabilistic algorithm, and the default tractography analysis parameters provided the best overlap with the reference AR.

### Interim summary

Starting from the same data and the same low-level diffusion model, the use of different MRI acquisition parameters, tractography algorithm and tractography parameters lead to very different tractography reconstructions of the AR. Overall, probabilistic reconstructions resulted better in terms of topographical AR accuracy, showing no significant effects of Dice overlap as function of the parameters manipulated. In addition, probabilistic algorithms can recover the AR even at low b-values (e.g., b = 1,000 *s*/*mm*^2^, commonly used in clinical protocols), for which the deterministic algorithm could not reconstruct any or only very few streamlines. Consistently, significant effects in the accuracy of AR reconstruction in comparison to the reference AR were found only for deterministic algorithms, for shell and angle threshold (respectively *p* = 0.0127 and *p* = 0.0327). Step size had minor effects on the AR reconstruction (Fig. 3). Overall, optimal results in terms of anatomical accuracy and correspondence to our AR reference where obtained for probabilistic tractography, using high b-values and default tractography parameters.

### Full group characterization and atlas reconstruction

Based on the optimization results, we chose the following parameters to reconstruct the AR from the entire dataset (34 subjects): probabilistic tractography algorithm, b-values = 10,000 *s*/*mm*^2^, and default tractography parameters (step size = 0.75 *mm*, angle = 45°).

The AR was successfully reconstructed in most of the subjects, correctly following macro-anatomical landmarks and showing a low number of false positive reconstructions. Streamlines correctly leave the postero-lateral part of the thalamus and move first in a lateral and then antero-lateral direction, to terminate in HG. We observed no streamlines erroneously following the inferior-superior direction of the external capsule fibres. However, we still observed high variability across subjects: two subjects showed no reconstructed streamlines in the right hemisphere, and in 6 subjects only few streamlines were visible in one hemisphere. This was confirmed by the coefficient of variability (*CV*, the standard deviation divided the mean) as measured for the left (LH) and right (RH) brain hemispheres for the extracted tracts’ volume (*CV*:*LH* = 0.58, *RH* = 0.69) and number of streamlines (*CV*:*LH* = *RH* = 0.93). Fractional anisotropy (FA) and apparent fibre density (AFD) mean values were also extracted for each reconstructed AR. FA values were very low for all subjects (<0.3), and showed less group variability (*CV*:*LH* = 0.08, *RH* = 0.28), whereas AFD showed considerable variability across the group (*CV*:*LH* = 0.55, *RH* = 0.65).

A lateralization index was computed for all the extracted quantitative measures (volume, number of streamlines, FA, AFD) in each subject. Results of the Wilcoxon signed-rank showed a significant (*α* = 0.01) degree of left lateralization for AFD (*p* = 4.89*E*-05), volume (*p* = 0.0012), and number of streamlines (*p* = 5.80*E*-05). FA values show a bilateral distribution in almost all subjects (31/34) (*p* = 0.014). Overall, only two subjects showed a degree of right lateralization (Table 1).

**Table 1:** Acoustic Radiation Hemispheric Lateralization.

AR hemispheric lateralization: subject proportion and p-value				
	Left	Bilateral	Right	p-value
Apparent fibre Density	19/34	14/34	1/34	*p* < 0.01
Fractional Anisotropy	2/34	31/34	1/34	*p* > 0.05
Tract Volume	17/34	16/34	1/34	*p* < 0.01
Number of streamlines	24/34	8/34	2/34	*p* < 0.01

The table reports the number of subjects that show leftward (*LI* > 0.2), bilateral(−0.2 < *LI* < 0.2), or rightward lateralization (*LI* < −0.2) in the different measures extracted from the acoustic radiation (apparent fibre density, fractional anisotropy, tract volume, and number of streamlines). The p-value of the hemispheric difference of these measures is also reported. *LI* = lateralization index (See Supplementary Fig. S1).

In order to exclude that the lateralization pattern in AR volume was driven by the volume of the ROIs used in the tractography reconstruction, we measured the Spearman correlation coefficient between the AR volume lateralization index and the HG and thalamus volume lateralization index. Results show no significant correlation for the AR and thalamus volumes (Rho = 0.15, p = 0.386) or AR and HG volumes (*Rho* = 0.34, *p* = 0.043).

Given the possible implication of the AR in speech comprehension processing, and the association between language and the left hemisphere, we also investigated the correlation between the AR volume and the volume of the arcuate fasciculus (AF). The AF was virtually dissected in each subject and Wilcoxon signed-rank results showed a significant difference in volume across the two hemispheres (*p* = 5.64*E*-06,*α* = 0.01), that was left lateralized (*LI* > 0.2) in 13/34 subjects, and bilateral in 21/34 subjects. In none of the subjects the AF was right lateralized. However Spearman’s correlation coefficient between AR and AF lateralization indices did not result significant (*Rho* = 0.31, *p* = 0.074).

**Figure 4:**
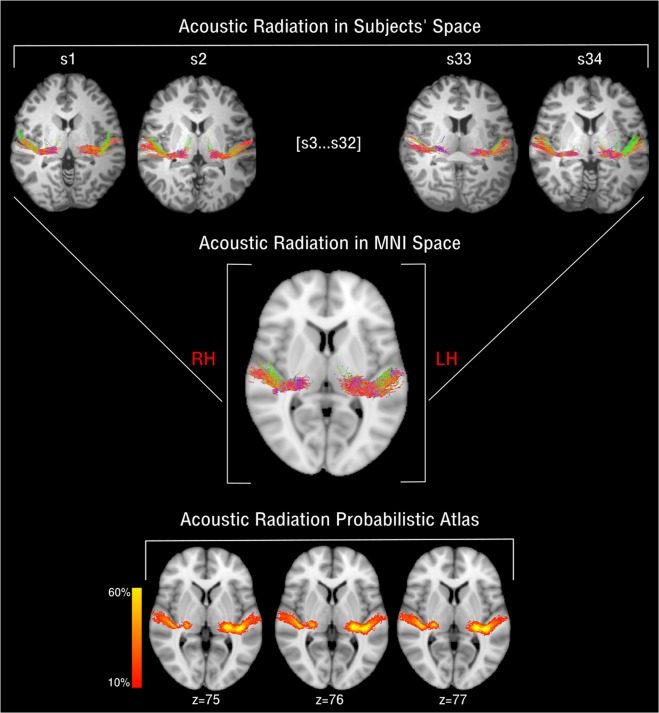
Atlas of the Acoustic Radiation. The figure shows the schematic pipeline for the construction of the AR atlas for the group of 34 subjects (s1, …, s34). As a first step, subject’s specific reconstructed AR streamlines (top row) are non-linearly registered to the MNI152 space (RH/LH: left and right hemispheres). Tractography reconstructions are then transformed to track density images (TDI), summed up and divided by the number of subjects and shown as a coloured proportion scale. The final AR atlas in MNI space is shown (bottom row).

**Figure 5:**
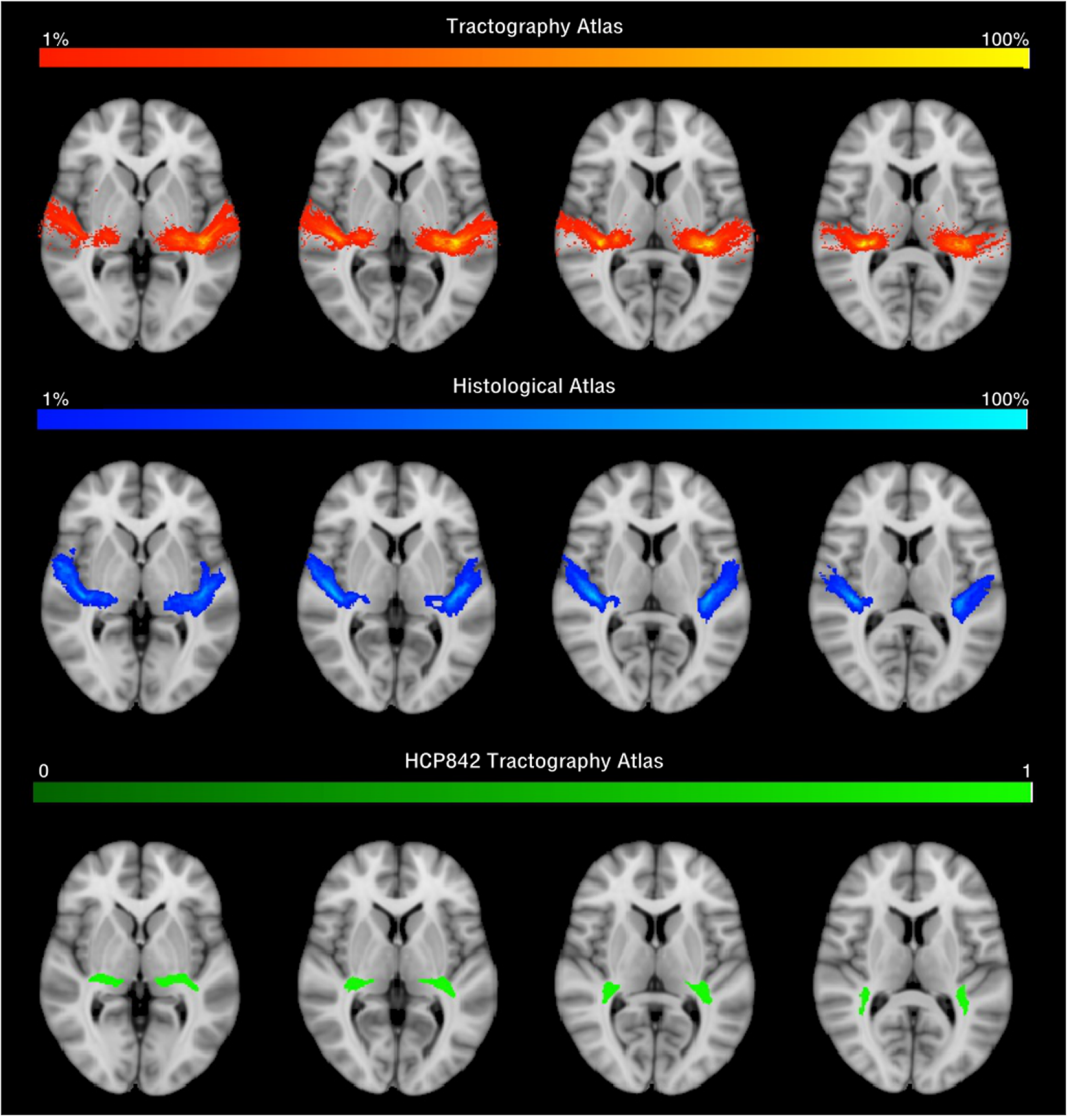
Comparison of acoustic radiation atlases. The figure shows the HCP tractography-based atlas reconstructed developed in this study (first row), the post-mortem histological atlas as reconstructed in^6^ (second row), and the tractography AR atlas as reconstructed in^20^ (third row). The three AR atlases are displayed on MNI space overlaid on the same T1 structural reference. The colour scales for the first two rows represent the percentage of subject presenting AR streamlines in that voxel. The third atlas is not probabilistic and it is binarized, showing either the presence or the absence of the AR in that voxel.

As a last step, we built a probabilistic tractography AR atlas in MNI space using the tractography reconstructions from the 34 subjects (Fig. 4). We then compared the results of this atlas with two publicly available AR atlas: a post-mortem histological atlas^6^ and a more recent tractography atlas^20^ (Fig. 5). The atlases did not show high spatial correspondence on the right (ARR) and left (ARL) brain hemispheres (Dice histological atlas: *ARR* = 0.43, *ARL* = 0.45; Dice tractography atlas: *ARR* = 0.123, *ARL* = 129). While showing good overlap at the thalamus origin of the bundle, the atlases diverge most near to the cortical termination of the AR in the Heschl’s gyrus.

## Discussion

This study focuses on the tractography reconstruction of the human acoustic radiation, a white matter bundle that, with few exceptions^4,8,21^, is largely absent from tractography studies and atlases. This is probably due to the fact that the location and anatomy of this bundle meet the current limits of diffusion-based tractography.

We investigated the effect that different diffusion MRI acquisition and tractography parameters have on the final 3D reconstruction of this bundle. The main findings of the study are: (i) higher b-values (≥5,000 *s*/*mm*^2^) and more DW directions (≥128) increase the accuracy of the AR reconstruction for both probabilistic and deterministic tractography; (ii) only probabilistic tractography can reconstruct the AR at low b-values (≤3000 *s*/*mm*^2^); (iii) the AR reconstruction remains challenging and highly variable across subjects; and (iv) the AR volume was found to be predominantly left lateralized. Additionally, we built a probabilistic tractography-based atlas of the AR reconstructed from 34 healthy subjects which we make open access (https://neurovault.org/).

The choice of acquisition parameters (b-value and number of diffusion directions), tractography algorithm (probabilistic and deterministic), and tractography parameters (angle threshold and step size) differently affected the sensitivity and specificity of AR tractography results. Previous studies investigated the dependence of tractography outcomes on user-defined parameters^14–17^ and agreed that no absolute optimal set of parameters for all white matter bundles exists. The choice of parameters strictly depends on the white matter region of interest and the specific anatomical features of the white matter bundle that is investigated.

The major effect in AR reconstruction accuracy is given by the higher b-value, for both probabilistic and deterministic tractography. This is most likely due to the higher angular resolution that can be achieved at higher b-values and allows to better resolve crossing configurations inside the voxel^22^. This has great impact on the AR, being a relatively small pathway, compared to other white matter bundles, that crosses major projection systems (e.g. internal capsule) at an almost orthogonal angle. This results in an under-representation of its signal in the diffusion data and consequent small volume fractions in the reconstructed FODs. For low b-values (1,000 and 3,000 *s*/*mm*^2^), no, or only a few (<5), streamlines were reconstructed using the deterministic approach. For higher b-values (5,000 and 10,000 *s*/*mm*^2^), more streamlines belonging to the AR could be reconstructed. However, their anatomical accuracy remained lower than probabilistic results. Visual inspection of tractograms suggests there may be false positive reconstructions at the level of the thalamus, that are not present in probabilistic counterparts (Fig. 1). Probabilistic tractography always allowed the reconstruction of AR streamlines, even at low b-values. However, at b = 1,000 *s*/*mm*^2^ many of these reconstructed streamlines constituted false positive reconstructions (Fig. 1). Most streamlines erroneously followed the course of the external capsule on the inferior-superior axis, as previously noticed^9^. These false positive artefacts are no longer visible at higher b-values (>5000).

Overall, deterministic results were found to be less robust than probabilistic results relative to the parameters used. We believe this is related to the very nature of the algorithm. At every voxel, the deterministic algorithm follows the direction of the extracted maxima of that FOD lobe. This makes the algorithm extremely sensitive to parameters that affect the estimation of the main peak (acquisition parameters) or the propagation along that direction (tractography parameters). Probabilistic tractography however draws samples from the distribution of directions around the main peak, treating the ODF as if it were a probability mass function. This inherently introduces a higher degree of tolerance, given the diminished dependency on the FOD maxima. Accordingly, significant effects in the Dice coefficient were shown for the b-value and angle threshold only for deterministic reconstructions.

In summary, we found that in terms of comparison with the reference AR, the optimal parameter choices for the atlas reconstruction were: probabilistic tractography based on the *b* = 10,000 *s*/*mm*^2^ data using the default tractography parameters (angle threshold = 45°, step size = 0.75 *mm*). Increasing the angle at high b-values only increased the false positive reconstructions and decreased anatomical accuracy, as previously shown^15^. The effects of increasing the step size were almost negligible (Fig. 2), although making tractography smoother, but when using very low step sizes (<0.5 mm), reconstructions were very noisy, as previously reported^17,23^. However default parameters were not optimal for different configurations. For example, for low b-values (1,000 and 3,000 *s*/*mm*^2^) and deterministic tractography results were improved by increasing the angle threshold up to 60°. Conversely, for low b-values and probabilistic tractography, results could be improved when small angle thresholds were used in the reconstruction (20°), obtaining results comparable to high b-values in terms of anatomical accuracy (Fig. 3). In this case, the low angle threshold is mainly filtering out false positive artifacts in the central part of the AR, at the level of the crossing with the internal capsule. We do not believe these streamlines correspond to anatomically plausible fibres, as they are absent in post-mortem dissections of the AR^9^. Knowing this, might help obtaining more accurate AR reconstructions when using clinical diffusion MRI protocols.

The reconstructions of the AR using the optimal parameters showed a high level of inter-subject variability. For example, the number of reconstructed streamlines ranged from 0 to 631, and the tract volume from 0 to 6834 *ml*. Inter-subject variability in tractography reconstructions has been previously investigated and can be related to individual anatomical differences and concomitant methodological limitations like uncertainty in fibre orientation estimation due to noise, head movements, and partial volume effects^5,24–26^. In addition, individual anatomical variability of the seed and target ROI might also contribute to AR reconstruction variability. Both the Heschl’s gyrus (HG) and the MGN, are subject to extreme anatomical variability across individuals and hemispheres, both in location and size^6,7^. The HG is characterized by very different configurations across subjects, and even across hemispheres^27^: narrower and smaller configurations might make the tractography reconstruction more challenging. Further studies might investigate the relationship between HG anatomical configurations and AR reconstructions.

The group variability of metrics derived from the AR was lower for FA values, in agreement with previous studies^25^. However, FA values were overall very low (*min* 0.17–*max* 0.32), even lower than previous studies looking at FA values in the AR^8^, and lower than what it is usually considered to be the threshold to differentiate grey and white matter (0.2). This is most likely related to the dense fibre crossing of this region. This poses limits on the possible uses of this metric to evaluate this specific white matter bundle, and makes the implementation of more advanced quantitative measures necessary^28,29^. In this study we also evaluate the AFD of the AR, as implemented in MRtrix3. Similarly to tract volume, AFD showed high variability across subjects. This measures has been recently introduced^28,30^ and used in clinical populations^31^. However, at this point we cannot put our AFD findings in perspective with the literature because to the best of our knowledge no studies have investigated AFD variability across subjects and white matter bundles.

Significant hemispheric difference was found in the AR reconstruction of the 34 subjects, showing a strong left lateralization (Table 1). These results are in contrast with a previous histological study that found no significant asymmetry in the volume of the AR in 10 *post-mortem* brains^7^. The small number of subjects we analysed in this work limits the certainty of the results. More studies investigating this asymmetry in different populations, and using different methods are needed. Even if we did not find a significant correlation in hemispheric lateralization between the arcuate fasciculus and the AR, our findings open interesting avenues for investigating the well-known relationship between the left hemisphere and language processing^5,32,33^.

The comparison between the AR probabilistic atlas constructed in this study and two publicly available AR atlases^6,20^ showed spatial overlap discrepancies, especially at the termination site on the HG. This difference was more evident when comparing our atlas with the tractography based atlas from Yeh *et al*. For both atlases the higher overlap is shown in the first part of the bundle, at its stemming from the thalamus. Further studies are needed to understand whether this difference is due to anatomical variability in the AR or to the different methodologies used and their possible limitations. As in all diffusion MRI tractography studies, the results obtained in this study are restricted to the parameters used for MRI acquisition, diffusion modelling and tractography reconstruction. Different acquisition protocols, low level models, tractography parameters, and region of interests, could lead to different findings. In this study, for example, we did not investigate the effects of MRI spatial resolution (kept at 1.5 *mm* isotropic), which may affect tract reconstruction and diffusion metrics estimation^34,35^. However, previous studies showed that varying spatial resolution, up to certain limits (<2.5 *mm* isotropic), did not affect tractography reconstructions as much as changes in angular resolution^36^. Also, we did not specifically address whether it was the number of DW directions or the b-value that affected the reconstruction the most. Given that optimal angular resolution might be obtained by a combination of the two, it is possible that the same results are obtainable for lower b-values and more DW directions^36,37^. Our findings suggest that anatomical reconstructions of the AR are more accurate when using the high b-values of the HCP data. The acquisition of such high b-values has well known challenges in terms of SNR. These may be at least partially addressed by the technological advances that make possible the use of stronger and faster magnetic field gradients in new generation MRI systems, such as those used by the Human Connectome^38^. Further experiments are needed to evaluate the degree to which atlases constructed from HCP data may used to drive the reconstructions of tractograms derived from diffusion MRI data acquired with less performing gradients. In this study the accuracy of the tractography reconstructions was evaluated by comparing them to subject’s specific AR references computed in a previous study^9^ and manually filtered to eliminate false positive artefacts. This allowed us to evaluate the results on an anatomical qualitative and quantitative basis: increased number of streamlines, or increased volume, most often are weakly related to the anatomical accuracy of the reconstruction. However, our reference had clear limitations. Being tractography-based itself, it suffers from the same limitations of the other reconstructions. The manual filtering was aimed at eliminating most false positive of the reconstruction. However, false negatives in the initial reconstruction may bias comparison with the other tractograms. Moreover, these references were reconstructed using probabilistic tractography, which might have favoured probabilistic tractograms in the comparison. Future studies might improve the quality of this comparison, using references obtained with different methods, such as more advanced *post-mortem* validation techniques^39^.

The Dice scores obtained in the study were overall low, and lower than other reports on different tracts^40^. However, those studies used the Dice coefficient to quantify test-retest reproducibility of the same tract over two different scans, thus making the comparison with our study not straightforward. In a previous human brain *post-mortem* dissection study, we defined three sub-components of the AR bundle: the *genu*, the stem, and the fan^9^. Future steps of this study will include applying this knowledge on the reconstructed AR, by isolating the AR stem. Previous studies have shown that performing tractography from the stem improves the final tractography reconstruction, by minimizing the constrained posed by cortical terminations^41^. We think this might help on different fronts in the tractography reconstruction of the AR: we would avoid seeding from the thalamus, which is well-known to be challenging in tractography^15^ and we might increase the number of streamlines that successfully reach the cortex on HG, given that the AR stem is lateral to the intermingling fibres of the internal capsule.

This study presents the first human tractography atlas of the acoustic radiation from a population of 34 young healthy subjects. The atlas was constructed using high quality MRI data from the Human Connectome Project. The acoustic radiation reconstruction was optimized with a systematic evaluation of MRI acquisition and analysis parameters using as reference reconstructions validated from an *ex-vivo* dissection study from our group^9^. The optimized reconstruction parameters for the acoustic radiation and the atlas may be used in future studies interested in identifying and characterizing the acoustic radiation both in health and in disease. The *in-vivo* reconstruction of this bundle would help understanding its involvement in cognitive deficit related to music and language processing, and in more general hearing disorders. Congenital and early deafness studies have shown differences in grey and white matter regions of the auditory system^42,43^, but to the best of our knowledge, no specific study on the AR in deaf subjects has been conducted to date. Furthermore, considering the evolution of brain surgery towards functional tailored resections, successful AR tractography reconstruction can be crucial for surgical planning^44^, in cases of temporal lobe resection, or even pre-operative assessments for cochlear implantation^45^.

## Methods

Pre-processed diffusion and structural MRI data of 35 healthy adult subjects (16 females, mean age: 31.1 years) provided by the MGH-USC Human Connectome project were analysed. In a previous study by our group^9^ we investigated the human AR morphology by comparing post-mortem micro-dissections and AR tractography reconstructions using a subset of the same dataset and default Mrtrix tractography parameters. That study raised the question of whether an optimal set of tractography parameters would exist for AR reconstruction and how this would change for different acquisition parameters. In this study a subgroup of 5 subjects is used to investigate the effects of MRI acquisition (b-factor and number of diffusion directions), tractography method (probabilistic or deterministic), and tractography parameters (step size, angle threshold) on AR tractography reconstruction. Optimal parameters are used to reconstruct the AR in all the remaining subjects and build a tractography atlas of the AR.

### Diffusion data processing

Diffusion weighted data was pre-processed as previously described^46^. The data is constituted by a multi-shell acquisition (b-factor = 1,000/3,000/5,000/10,000 *s*/*mm*^2^) for a total of 552 directions, of which 512 diffusion weighted (DW) and 40 non-DW volumes (b = 0), at a spatial resolution of 1.5 *mm* isotropic. In order to investigate the effect of acquisition parameters, the four shells were divided and separately analysed in MRtrix3^23^. For each b-value we extracted all the corresponding diffusion volumes, without varying the number of diffusion encoding directions 2. For each shell, bias field correction and global intensity normalisation were performed and an average response function was calculated from the subjects’ specific response functions. Fibre orientation distribution functions (fODF) were then recovered for each subject using constrained spherical deconvolution (CSD) on the basis of the shell-specific average response function. The tensor model was also fit to the data and fractional anisotropy (FA) maps were extracted.

### Structural data processing

The T1 weighted structural MRI of each subject was linearly registered through affine registration to the diffusion space of each subject in FSL (https://fsl.fmrib.ox.ac.uk/fsl, version 5.0). For better co-registration results, the up-sampled FA map (1 × 1 × 1 *mm*^3^) was first registered to the T1, and then the inverse transformation was applied to the T1 image. The registered T1 was then segmented in FSL^47,48^ to obtain white matter, grey matter, and CSF partial volume estimate (PVE) maps, and subcortical nuclei masks. This information was combined to create a five-tissue-type image to be used for anatomically constrained tractography (ACT)^49^.

### AR dissection protocol

The AR was reconstructed using the same protocol as in^9^. The right and left thalami as segmented in FSL were used as seeding regions of interest (ROI) to initiate tractography of the AR (see Supplementary Fig. S2). The Heschl’s gyrus (HG) was manually segmented in each subject and brain hemisphere and used as a target ROI for the AR tractography reconstruction. The HG is defined anteriorly by the transverse sulcus (TS) of the temporal lobe, which unites medially to the circular sulcus of the insula, and posteriorly by the Heschl’s sulcus (HS). There might be a second HS (or *sulcus intermedius*) if two HG gyri are present^27,50^. In every subject the HG was first identified on the sagittal slice and its borders were marked for every slice from the first medial slice, where HG becomes visible as a protrusion of the STP, to the more lateral slice where it disappears. Once defined on the sagittal slice its shape was followed and refined on the axial slice, carefully following the grey matter near to the transverse sulcus and Heschl’s sulcus. As a last step the coronal plane was checked. Both grey matter and white matter were included in the segmentation, and, if present, the second gyrus was included as well. The same ROI was drawn five times for each subject and each hemisphere and accuracy was then measured by calculating the Dice coefficient between successive drawn ROIs, and mean and standard deviation were calculated (mean and sd) (see Supplementary Fig. S3). The Dice coefficient is calculated as $(overlap=\frac{2(mas{k}_{A}\cap mas{k}_{B})}{mas{k}_{A}+mas{k}_{B}})$^51^. A Dice coefficient of 0.0 corresponds to no overlap between the two segmentations, while a Dice coefficient of 1.0 corresponds to identical regions of interest.

### Optimal AR tractography reconstruction

A subgroup of 5 subjects (MGH_1001, MGH_1002, MGH_1003, MGH_1004, MGH_1005) was used to investigate the effects of MRI acquisition and tractography parameters on AR tractography reconstruction. For each one of these subjects, the left and right hemisphere AR was reconstructed using one set of acquisition and analyses parameters as defined in Table 2. In other words, the 72 different AR reconstructions arose from 4 possible single-shell acquisitions, 2 tractography algorithms (deterministic and probabilistic) and 9 combinations of step-size and angle thresholds (default for both, default step-size with 4 angle parameters, default angle with 4 step sizes). The maximum streamline length (80 *mm*)^9^ and the number of seeds per voxel (*n* = 2000) were kept constant 2). Seed points were generated at random by uniform sampling of the seed ROI for both probabilistic and deterministic algorithms^23^. This number of seeds was chosen to limit false-negative results related to poor sampling (see Supplementary Fig. S5). Both probabilistic and deterministic tractography algorithms were initiated from every voxel of the thalamus and the manually segmented Heschl’s gyrus was used as inclusion ROI (section: AR dissection protocol). The algorithm was instructed to stop at the grey matter/white matter interface, as defined by the structural T1 data. Overall, this gave 72 different AR tractography reconstructions per subject, per brain hemisphere.

**Table 2:** MRI acquisition and tractography parameters.

Shell (s/mm^2^) and	1000, 3000, 5000, 10000
Number of DW Directions	64, 64, 128, 256
Tractography algorithm	Probabilistic, Deterministic
Angle thresholds per step-size = 0.75 mm	20, 30, 45, 60, 80°
Step sizes per angle threshold = 45°	0.25, 0.50, 0.75, 1, 1.25 *mm*
Maximum streamline length	80 *mm*
Number of seeds per voxel	2000

The table shows the different MRI acquisition and tractography parameters used to evaluate the reconstruction of the human acoustic radiation.

The effects of MRI acquisition and tractography parameters on AR reconstructions were evaluated by measuring the spatial overlap with a subject’s specific reference AR reconstruction. The reference AR was obtained by using the the multi-shell tractography reconstructions of the same subjects obtained in the previous study^9^ after these have been manually inspected and filtered (edited) by the neuro-surgeon (author S.S) that performed the *post-mortem* dissections (see Supplementary Fig. S4).

One subject (subject MGH_1003) was excluded from the reference generation, since both the automatically reconstructed and the filtered tractograms resulted in too few streamlines to allow for meaningful comparisons (<10 streamlines; average number of streamlines for the other subjects = 600). To quantify the anatomical correspondence between the reference tractograms and the reconstructed tractograms a binary mask was created for all voxels intersected by at least one streamline. From these binary masks the volumetric overlap was quantified as the Dice similarity coefficient. The Dice coefficient has been used before in tractography studies to assess reconstruction reproducibility^36,52,53^. In this study the Dice coefficient is used as a measure of 3D volume overlap between the reference AR and the single shell AR reconstructions at different analysis parameters. Acquisition and analysis parameters giving the highest Dice coefficient values were regarded as optimal.

### Full group acoustic radiation tractography reconstruction

The set of acquisition and tractography parameters that better reconstructed the profile of the AR were then applied to the complete dataset (34 subjects): b-factor = 10000 *s*/*mm*^2^, probabilistic tractography, 0.75 *mm* step size, 45° angle threshold. One subject was excluded (MGH_1020) because it had an incomplete acquisition (482 volumes instead of 552 b = 10000 *s*/*mm*^2^ volumes).

Hemispheric tract-specific AR measures were then extracted (tract volume, number of streamlines, FA, AFD). FA values were extracted from the FA scalar map computed for b = 1000 *s*/*mm*^2^, to avoid FA confounding at high b-values^54^. Apparent fibre density (AFD) was computed using the *afdconnectivity* command in Mrtrix3. At high b-values (as used in this study) the integral for a particular FOD ‘lobe’ is considered proportional to the intra-axonal volume of axons associated with that lobe^30^. Compared to FA which is voxel-based, AFD is tract-specific. Only the lobes of the FOD that are associated to AR streamlines are used to compute the AFD. This makes AFD a more specific measure, less prone to complex fibre configuration biases. Coefficients of variation (CV) were computed for each extracted measure and each subject in both hemispheres. CV is defined as the ratio of the standard deviation *σ* to the mean of the population. A laterality index (LI) was also computed (*LI* = *L* − *R*/*L* + *R*, R: right, L: left) on the tract-specific metrics to investigate AR hemispheric asymmetry. The LI ranged from −1 (completely righ–lateralized) to +1 (completely left–lateralized). In concordance with prior studies, bilateral representation was defined in the −0.2 to +0.2 range^55^.

### Arcuate fasciculus reconstruction

In order to investigate the relationship between the AR lateralization and language we also reconstructed the arcuate fasciculus (AF), a tract that is associated to language processing and is often found to be left-lateralized^56^. The AF was virtually dissected in Trackvis (www.trackvis.org) by author C.M. starting from the whole brain tractogram. The whole brain tractogram was obtained using the same optimal parameters used for AR reconstruction: b-factor = 10000 *s*/*mm*^2^, probabilistic tractography, 0.75 *mm* step size, 45° angle threshold. To dissect the AF two ROIs were used: one encompassing the white matter of the superior temporal gyrus and one near the pars opercularis. Only streamlines passing through both ROIs were retained. Tract volume was extracted for each bundle.

### Acoustic radiation atlas construction

The AR atlas was constructed using the optimal AR reconstructions from the 34 subjects. We first computed the warping images between the diffusion space of each participants and the standard MNI space through a two step diffeomorphic registration performed in ANTS software^57^. The up-sampled subject’s FA map (1 × 1 × 1 *mm*) was first registered to the subject’s T1; the subject’s T1 was then registered to the MNI_152_1 mm space. The inverse warps were then concatenated and applied to the reconstructed streamlines of each participant through the *tcknormalize* command implemented in MRtrix3. We then computed the tract density image (TDI) of each streamline AR bundle in MNI space and binarized it in MRtrix3. All binary images were then summed together and divided by the number of subjects to build the final AR atlas. The tractography reconstructions were not computed in standard MNI space in an attempt to preserve the individual anatomical information. This could be especially important in the case of the AR because of the great structural variability of the Heschl’s gyrus (HG) across subjects and hemispheres. For this same reason we manually drew the HG segmentations in each individual subject. As a last step, we also measured the Dice spatial overlap between the AR tractography-based atlas and two publicly available atlases of the AR: a *post-mortem* histological atlas available in FSL^6^, and a more recent tractography-based atlas available in DSI Studio (dsi-studio.labsolver.org).

### Statistics

Statistical analyses were carried out in Python using Pandas and Scilpy frameworks. We tested our data for normality using the Shapiro Wilk test. With the exception of a few variables (AR streamlines count and FA, *p* < 0.01) data resulted normally distributed. However given the small sample size (*N* ≤ 34), we decided to use non-parametric statistics throughout the analyses. A Kruskall-Wallis non parametric test was performed to evaluate the effect of varying acquisition and tractography parameters on the Dice coefficient. Alpha level was set to 0, 05 given the small sample size (*N* = 4). The Wicoxon signed-rank test was used to look for differences in tract-specific metrics across hemispheres. Aplha level was set to 0, 01 in this case. To investigate the correlation between variables we measured the Spearman’s correlation coefficient setting alpha at 0, 01.

## Supplementary information

Supplementary Material

## Data Availability

The datasets generated during the current study are available in the https://NeuroVault.org repository (https://neurovault.org/collections/OIKTIIJG/^58^).
