# Supplementary material for: Diffusion-based tractography atlas of the human acoustic radiation
Source: Sci Rep. 2019 Mar 11;9:4046. doi: 10.1038/s41598-019-40666-8 (PMC6411970; doi:10.1038/s41598-019-40666-8)
Supplement: Supplementary file 1 — Supplementary Material [file 41598_2019_40666_MOESM1_ESM.pdf]

Supplementary Material

Diffusion-based tractography atlas of the human acoustic radiation

Chiara Maffei [1,2,] Silvio Sarubbo [3], Jorge Jovicich [1,4]

[1] CIMeC Center for Mind/Brain Sciences, Trento University, Trento, Italy
[2] Athinoula A. Martinos Center for Biomedical Imaging, Massachusetts General Hospital and Harvard Medical School
[3] Division of Neurosurgery, Structural and Functional Connectivity Lab (SFC-LSB) Project, "S.Chiara" Hospital, Trento APSS, Italy
[4] Department of Psychology and Cognitive Sciences, University of Trento, Italy

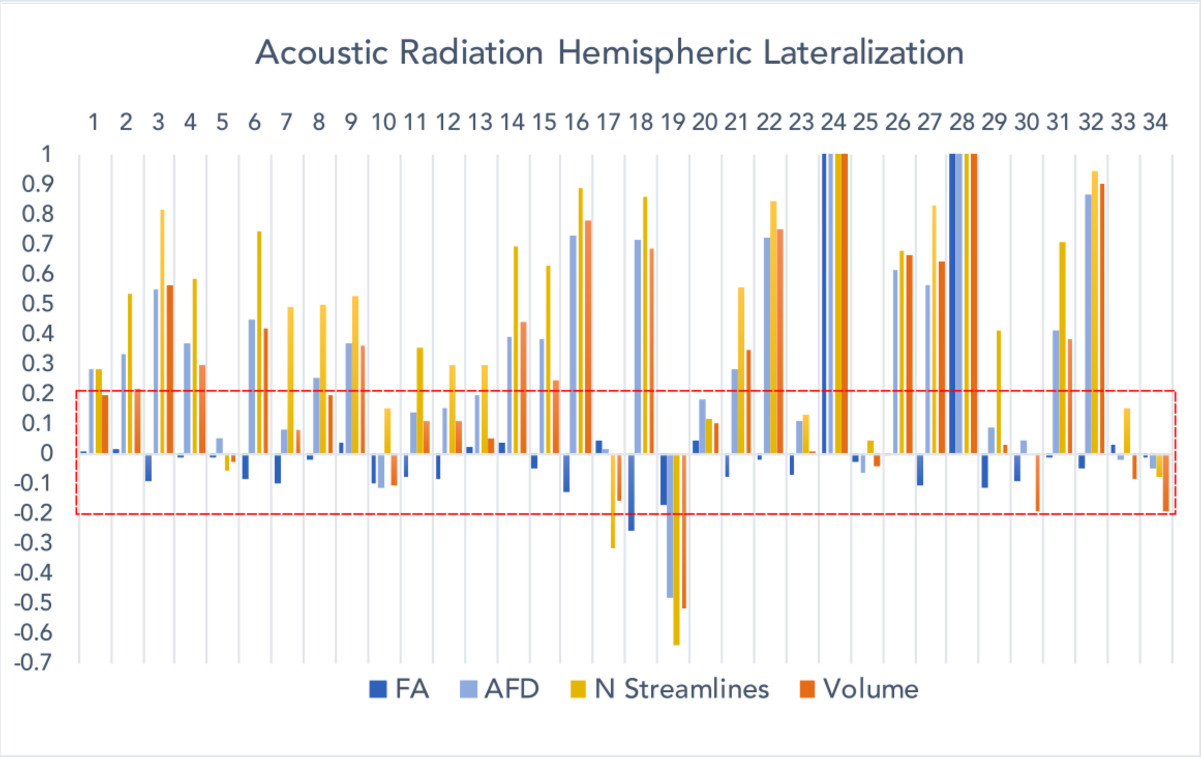

Supplementary Figure S1. **Lateralization Index of the Acoustic Radiation**. The graph reports the lateralization index (LI) of the AR for the 34 subjects computed from different metrics (FA: fractional anisotropy, AFD: apparent fiber density, number of streamlines and tractogram volume). The LI ranges from -1 (completely right$-$lateralized) to $+1$ (completely left$-$lateralized). Bilateral AR representation was defined in the -0.2 to $+0.2$ range, identified by the red dotted line. We found a significant ($\alpha=0.01$) degree of left lateralization for AFD ($p=1.5E-05$), volume ($p=4.3E-04$), and number of streamlines ($p=6.09E-04$).

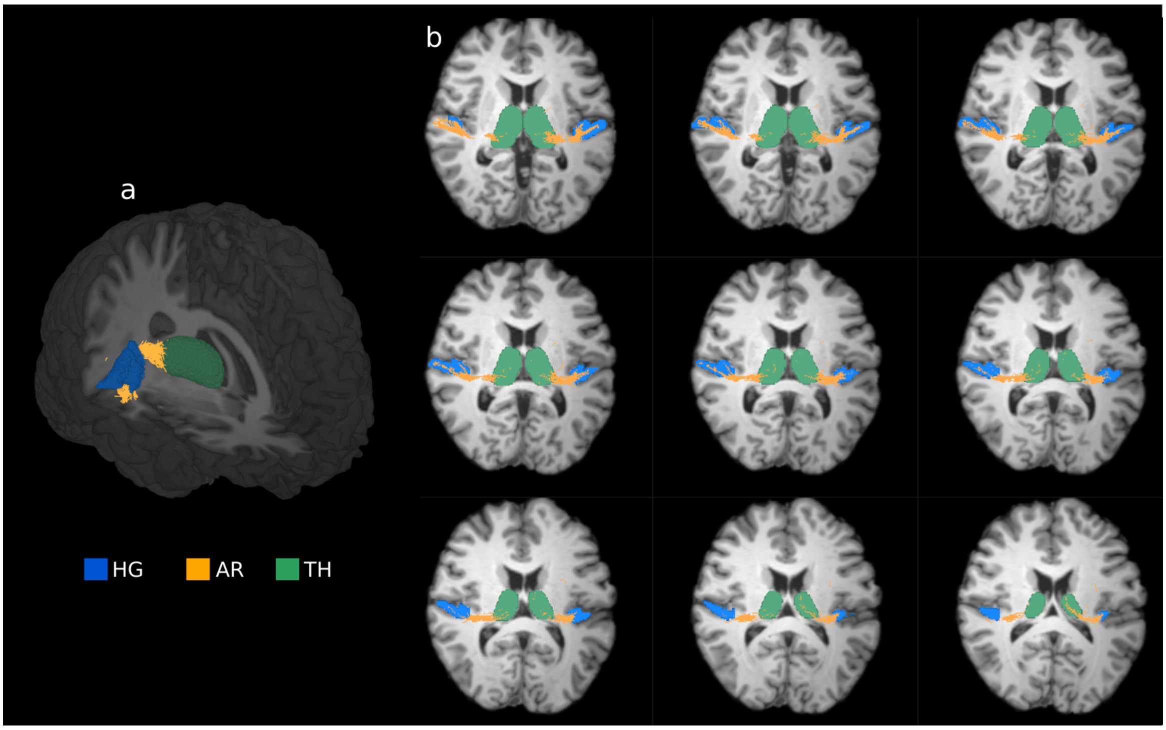

Supplementary Figure S2. AR dissection protocol. a) 3D rendering of the T1 weighted image of one representative subject together with the ROI used to reconstruct the AR and the AR tractography reconstruction. b) Light box axial view of the same subject. AR=acoustic radiation, HG= Heschl's gyrus, TH=thalamus.

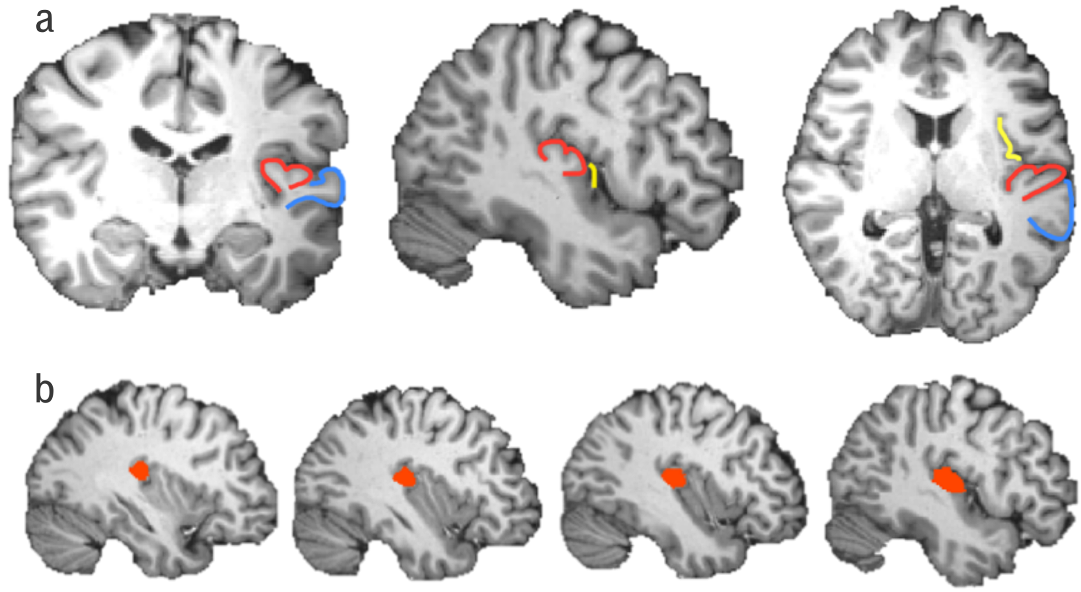

Supplementary Figure S3. Heschl's gyrus definition. T1 weighted image of one representative subject displaying boundaries of left HG (red), left planum temporale (blu) and left insular cortex (yellow). Images are displayed in radiological convention. b) The image shows the selection of HG on different sagittal slice, from the more medial to the more lateral one.

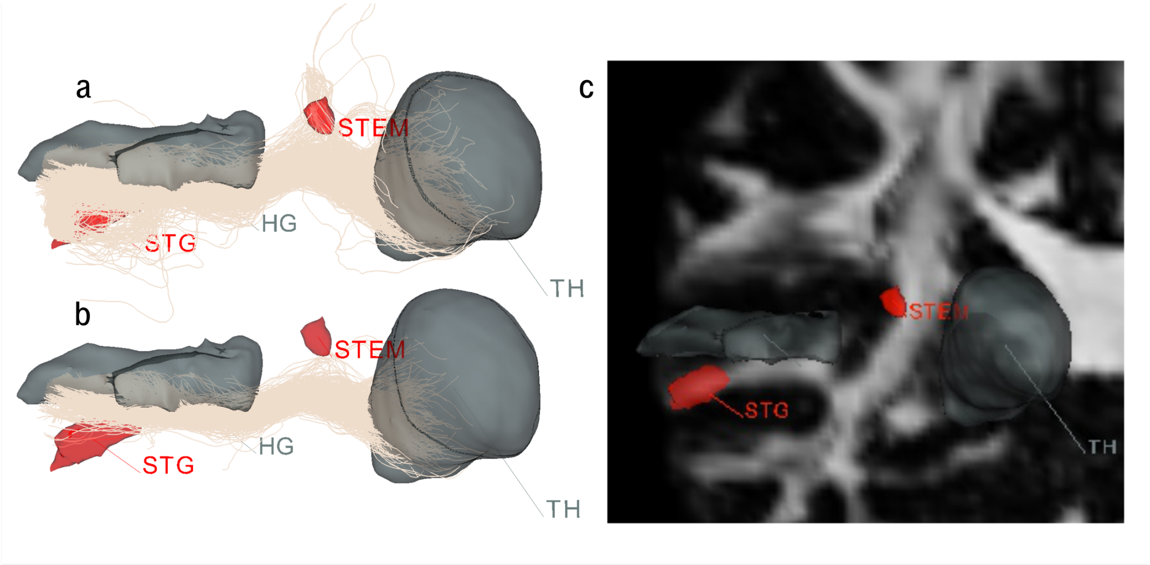

Supplementary Figure S4. Manual filtering of the AR. The figure shows the AR reconstruction before and after the manual filtering process. Most of the artefactual streamlines are situated at the level of the posterior dorsal portion of the external capsule, and at the level of the inferior portion of the posterior and middle thirds of the superior temporal gyrus. STG=superio temporal gyrus, HG=Heschl's gyrus, TH=thalamus, STEM=stem of the AR.

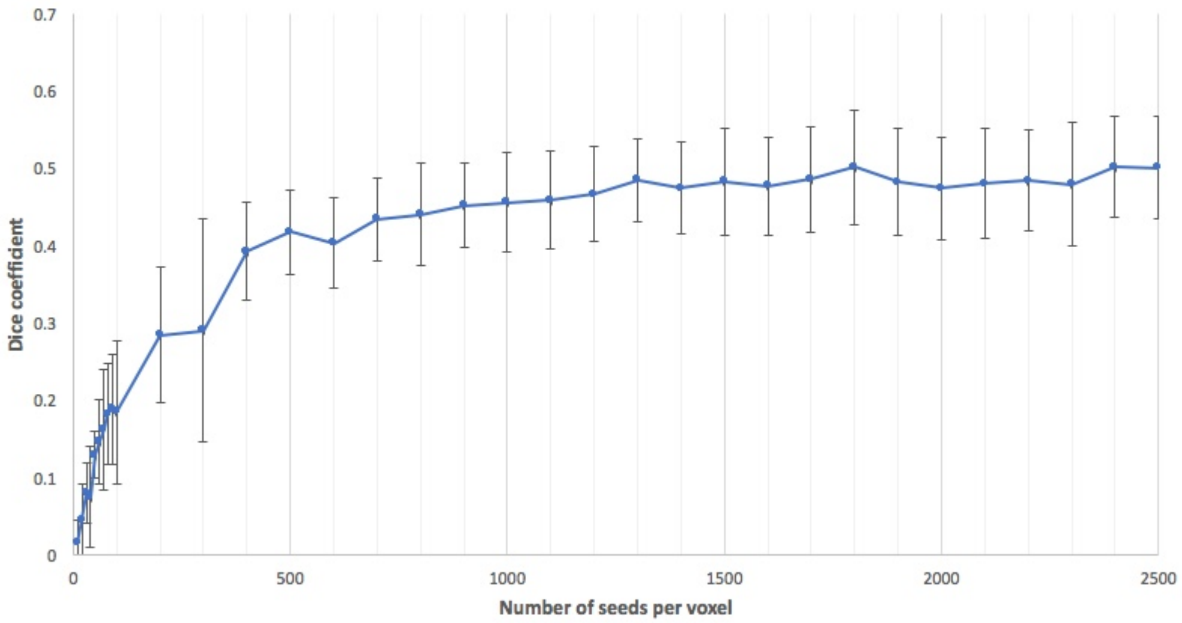

Supplementary Figure S5. Evaluation of voxel seed number. The graph shows the average Dice coefficient of spatial overlap between the edited AR reference and AR reconstructions using a range of voxel seed-number values for 3 subjects (0-100 with step=10; 100-2500 with step=100). AR reconstructions were obtained using the following parameters: probabilistic tractography, b=10000 s/mm^2, angle threshold = 45º, step size= 0.75. It can be seen that for a voxel seed-number greater than 700, the Dice score tends to stabilize (Kruskal-Wallis test, p=0.8399).
